# Direct Observation of Grain-Boundary-Migration-Assisted Radiation Damage Healing in Ultrafine Grained Gold under Mechanical Stress

**DOI:** 10.1021/acs.nanolett.3c00180

**Published:** 2023-04-14

**Authors:** Sandra Stangebye, Kunqing Ding, Yin Zhang, Eric Lang, Khalid Hattar, Ting Zhu, Josh Kacher, Olivier Pierron

**Affiliations:** † School of Materials Science and Engineering, Georgia Institute of Technology, Atlanta, Georgia 30332, United States; ‡ Woodruff School of Mechanical Engineering, Georgia Institute of Technology, Atlanta, Georgia 30332, United States; § Nuclear Engineering Department, University of New Mexico, Albuquerque, New Mexico 87131, United States; ∥ Sandia National Laboratories, Albuquerque, New Mexico 87185, United States; ⊥ Department of Nuclear Engineering, University of Tennessee, Knoxville, Tennessee 37996, United States

**Keywords:** in situ TEM nanomechanics, stress-assisted grain boundary migration, radiation damage healing, ultrafine grained Au

## Abstract

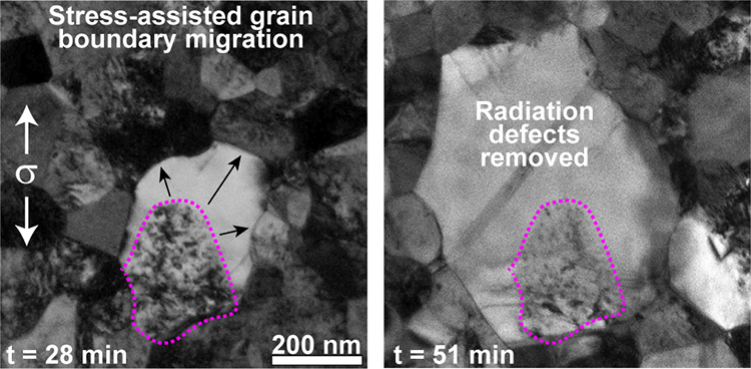

Nanostructured metals are a promising class of radiation-tolerant materials. A large volume fraction of grain boundaries (GBs) can provide plenty of sinks for radiation damage, and understanding the underlying healing mechanisms is key to developing more effective radiation tolerant materials. Here, we observe radiation damage absorption by stress-assisted GB migration in ultrafine-grained Au thin films using a quantitative *in situ* transmission electron microscopy nanomechanical testing technique. We show that the GB migration rate is significantly higher in the unirradiated specimens. This behavior is attributed to the presence of smaller grains in the unirradiated specimens that are nearly absent in the irradiated specimens. Our experimental results also suggest that the GB mobility is decreased as a result of irradiation. This work implies that the deleterious effects of irradiation can be reduced by an evolving network of migrating GBs under stress.

Material degradation resulting from irradiation^1−4^ is a challenge that plagues a variety of industries, including the nuclear energy sector^1,5^ and the aerospace industry.^6^ The property changes originate from displacement cascades caused by energetic particles such as neutrons, protons, and ions that lead to nanometer-sized defect clusters in the form of vacancy and interstitial loops, stacking-fault tetrahedra (SFT), or voids. In contrast to conventional nuclear materials,^7^ nanostructured metals and composites^8^ are currently investigated as a new class of radiation damage tolerant materials,^9^ given that interfaces, surfaces, and grain boundaries (GB) can serve as sinks for radiation-induced defects.^10,11^ Nanocrystalline (NC) and ultrafine-grained (UFG) metals have already shown increased radiation tolerance^12−15^ due to the high volume fraction of GBs.^14,16−21^ Molecular dynamics (MD) simulations suggest that GBs annihilate nearby vacancies by re-emitting interstitials^22^ or that regions of misfit within the boundary can be sites for interstitial and SFT absorption,^19^ both of which lead to the experimentally observed defect denuded zones on either side of a boundary.^20,23^*In situ* TEM irradiation experiments provide direct observation of defect coalescence and absorption at GBs^20,24−30^ which can be further analyzed to investigate the relative sink strength of various GBs.^31^ Numerous reports have shown that irradiation also causes GB migration (GBM) and grain coarsening,^29,32^ with MD simulations proposing that migration is a response to defect absorption.^33,34^ Additional reports have shown that radiation-induced GBM can remove SFT,^28^ indicating that a migrating GB serves as an effective sink for defects.

The aforementioned studies mainly focus on the role of GBs during irradiation in the absence of mechanical stress. MD simulations on bicrystals have highlighted that shear-coupled GB motion can remove intragrain SFT^35,36^ and partially dissolve voids,^37^ leading to an interstitial-loaded GB that can remove defects in its path.^38,39^ Given that stress-assisted GBM is a common deformation mechanism in NC and UFG metals,^40−43^ these materials may have an additional “self-healing” mechanism that could facilitate a further increase in radiation tolerance when subject to mechanical stresses.

In this work, we demonstrate that stress-assisted GBM is indeed an active healing mechanism at room temperature in irradiated UFG gold (Au) thin films through direct observation of migrating GBs absorbing irradiation-induced defects under stress. To that end, we utilize a quantitative *in situ* TEM nanomechanical testing technique based on a microelectromechanical system (MEMS) device (see Supporting Information for details of operation). In many cases, the defect-free regions can support prolonged dislocation glide and dislocation-dislocation interactions. Results also show a clear difference in GBM behavior with irradiated specimens exhibiting slower but steady GBM, whereas nonirradiated specimens experience rapid bursts in migration followed by stagnation.

The nonirradiated specimens in this study have been previously characterized and tested using a similar *in situ* TEM nanomechanical testing technique.^44−48^ A portion of the specimens were irradiated with 2.8 MeV Au^4+^ at room temperature at Sandia National Laboratories to ∼0.7 displacement per atom (dpa)^49^ (specimen fabrication and irradiation details are described in Supporting Information). The initial microstructure of the nonirradiated specimens (Figure 1a) shows that the majority of grains are defect free, with a few of the largest grains containing lattice dislocations and/or twin boundaries (arrowed). The initial microstructure of the irradiated specimens (Figure 1b) shows radiation damage within all the grains (damage seen at higher magnification in Figure 1c). Although not specifically characterized, the radiation damage is likely to be small dislocation loops or small SFT.^50^ Weak-beam dark-field (WBDF) was used to count the visible defects and the defect spacing *l* was estimated to be ∼15 nm (information on calculation provided in the Supporting Information). An indexed diffraction pattern for the irradiated Au (Figure S2) shows that the FCC structure is retained. Grain size (*d*) distributions show that radiation-induced grain growth results in a 33% increase in the average grain size from 142 to 189 nm, due to the near-total removal of grains smaller than 50 nm after irradiation (Figure 1d). This is consistent with *in situ* TEM irradiation studies that show that larger grains grow at the expense of smaller grains under irradiation alone.^29^Figure 1e shows the monotonic tensile stress–strain curves for a nonirradiated and irradiated specimen at a strain rate of ∼10^–4^ s^–1^.^62^ The monotonic response of the irradiated specimen shows evidence of brittle behavior with a linear elastic stress increase followed by failure after attaining the ultimate tensile strength (UTS) of 663 MPa. The nonirradiated counterpart yields at ∼480 MPa (0.2% offset), reaches an UTS of 520 MPa, which is followed by a gradual decrease in stress and eventual failure at plastic strain ε_*p*_ = 4.9%. The *post-mortem* fracture surface of the nonirradiated specimen (Figure 1f) indicates that the stress decrease after UTS is likely due to slight necking and stable crack growth promoted by the maximum shear stress approximately along the 45° direction with respect to the vertical loading axis. Similarly, Figure 1g confirms the brittle-type unstable crack growth that occurred in the irradiated film, with the fracture surface at about 90° from the vertical loading axis. The observed strengthening effect of the irradiated specimens is consistent with the *in situ* TEM observations of dislocation-radiation defect interactions, with the radiation defects serving as obstacles to dislocation glide.^51^ A detailed description and TEM based characterization of dislocation pinning is provided in Figure S4 (Movie S1). An additional example of intragranular plasticity is shown in Movie S2 and illustrates the significant restriction in dislocation glide due to radiation damage. In all of the irradiated specimens tested, there is no indication that defect-free channels form from repeated intragranular dislocation glide, which is a variation from what is typically observed in irradiated coarse-grained metals.^52,53^ This likely indicates that there is an insufficient number of passing dislocations to facilitate complete removal of radiation defects within localized regions.

**Figure 1:**
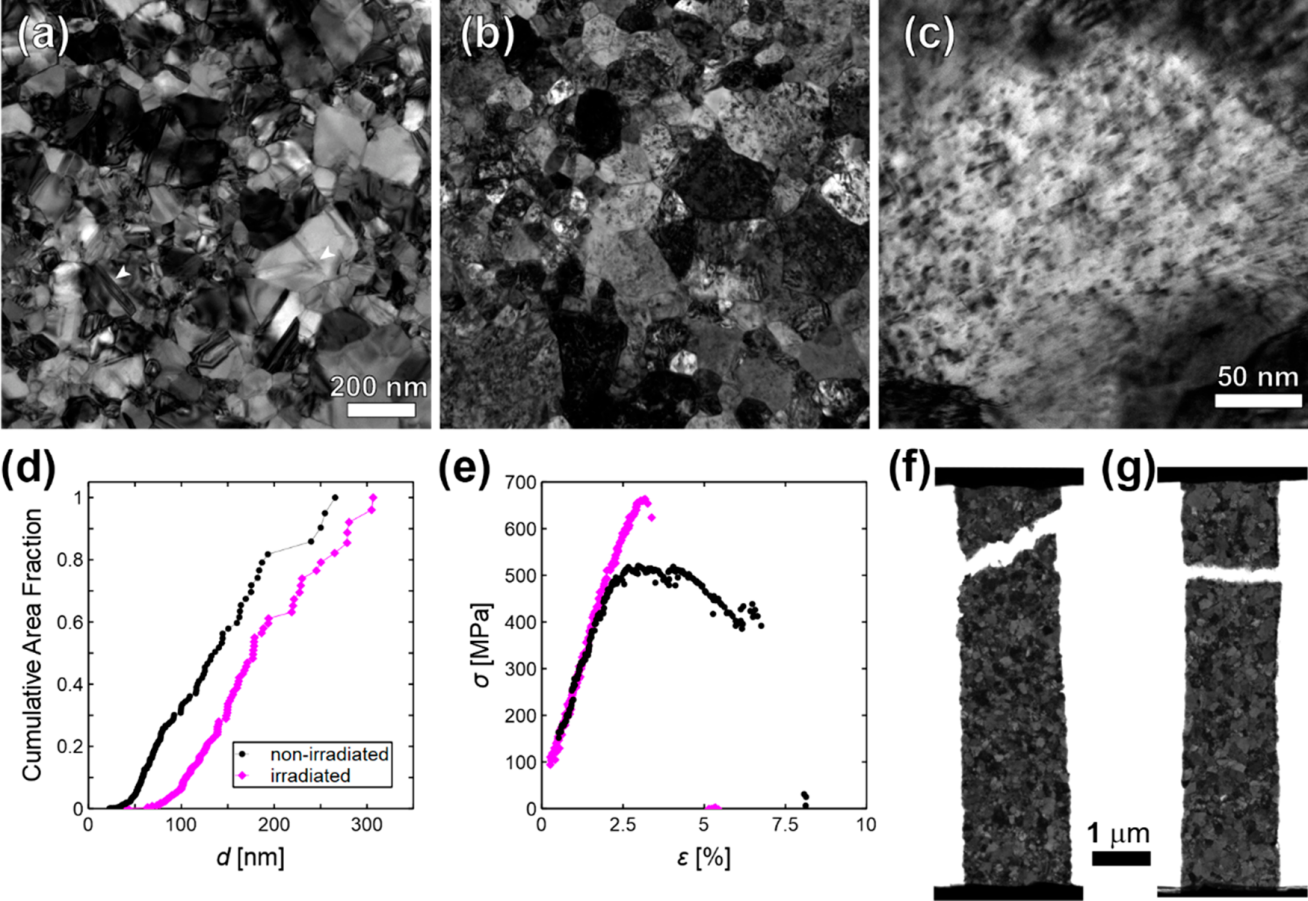
Initial microstructure and tensile properties of irradiated and nonirradiated UFG Au films. TEM micrographs of (a) nonirradiated Au film (white arrowheads identify nanotwins or dislocations within grains) and (b) irradiated Au film. Grain interiors contain radiation damage. Scale bar is the same as in part a. (c) Bright-field TEM micrograph of a grain interior of irradiated film to exhibit radiation damage. (d) Cumulative grain size distribution of nonirradiated (black circles) and irradiated (magenta diamonds) films prior to straining. (e) Stress–strain curves from *in situ* TEM tensile tests of nonirradiated and irradiated specimens. Both were conducted at a strain rate of ∼10^–4^s^–1^. *Post mortem* TEM micrographs of a (f) nonirradiated and (g) irradiated specimen tested under tension to show the differences in fracture surface.

In addition to restricted dislocation glide, stress-induced GBM is another active deformation mechanism in the irradiated specimens. Evidence of radiation damage removal via GBM is illustrated in Figure 2 where the outlined grain undergoes substantial grain growth during a stress-relaxation experiment. As the GB migrated, it absorbed the radiation defects in the neighboring grains, creating defect-free regions. This is clearly seen in Figure 2b where there is a region with radiation defects (where the original grain was) in addition to a region without any radiation damage as the GBs have migrated outward in the direction indicated by arrows. This image was taken after a 28 min-long series of stress-relaxation segments in which a maximum stress of 530 MPa was achieved (the stresses refer to the applied far-field values). Additional *in situ* TEM observations of this growing grain were made throughout another series of stress-relaxation segments, during which the GBs continued to migrate (Figure 2c–f). The outlined GB migrates at an average velocity of 0.03 nm/s (for σ < 530 MPa), not including the one instance of a rapid jump in GBM occurring at a maximum velocity of 34 nm/s during the transition from part b to part c of Figure 2 (Movie S3). This maximum velocity did not occur simultaneous to an applied stress increase, indicating a different factor contributed to the accelerated migration. In Figure 2c, a pinned dislocation is indicated by the arrowhead and 30 s later is depinned and glides unrestricted through the defect-free region (Figure 2d and Movie S4). For the transition in Figure 2c–e (Movie S4), migration velocities for each GB were determined by measuring the change in GB location in 30-s intervals (Figure S5). At an applied stress level of 550 MPa, the average GBM velocities range from 0.03 to 0.07 nm/s. Upon increase of the applied stress to 650 MPa (nearing the UTS of irradiated specimens, see Figure 1e), some average GBM velocities increase by more than 1 order of magnitude (to values ranging from 0.32 to 0.95 nm/s), suggesting that the local stresses may be significantly larger. After 51 min under tension, extensive GBM has led to a large defect free region that can support unrestricted dislocation glide and dislocation-dislocation interactions. This is seen in Figure 2f where there are multiple dislocations interacting with a partial dislocation within the defect-free region (Movie S5). The majority of the radiation defects remain within the original grain outline. This type of behavior is similar to grains distributed throughout the specimen gauge length; and in total, 14% of the specimen area was cleared of defects after this experiment. Additional details on this microstructural evolution and the accompanying movies are included in the Supporting Information. Figure S6 includes a magnified view of a migrating GB with evidence of disconnection glide within GB (Movie S6).

**Figure 2:**
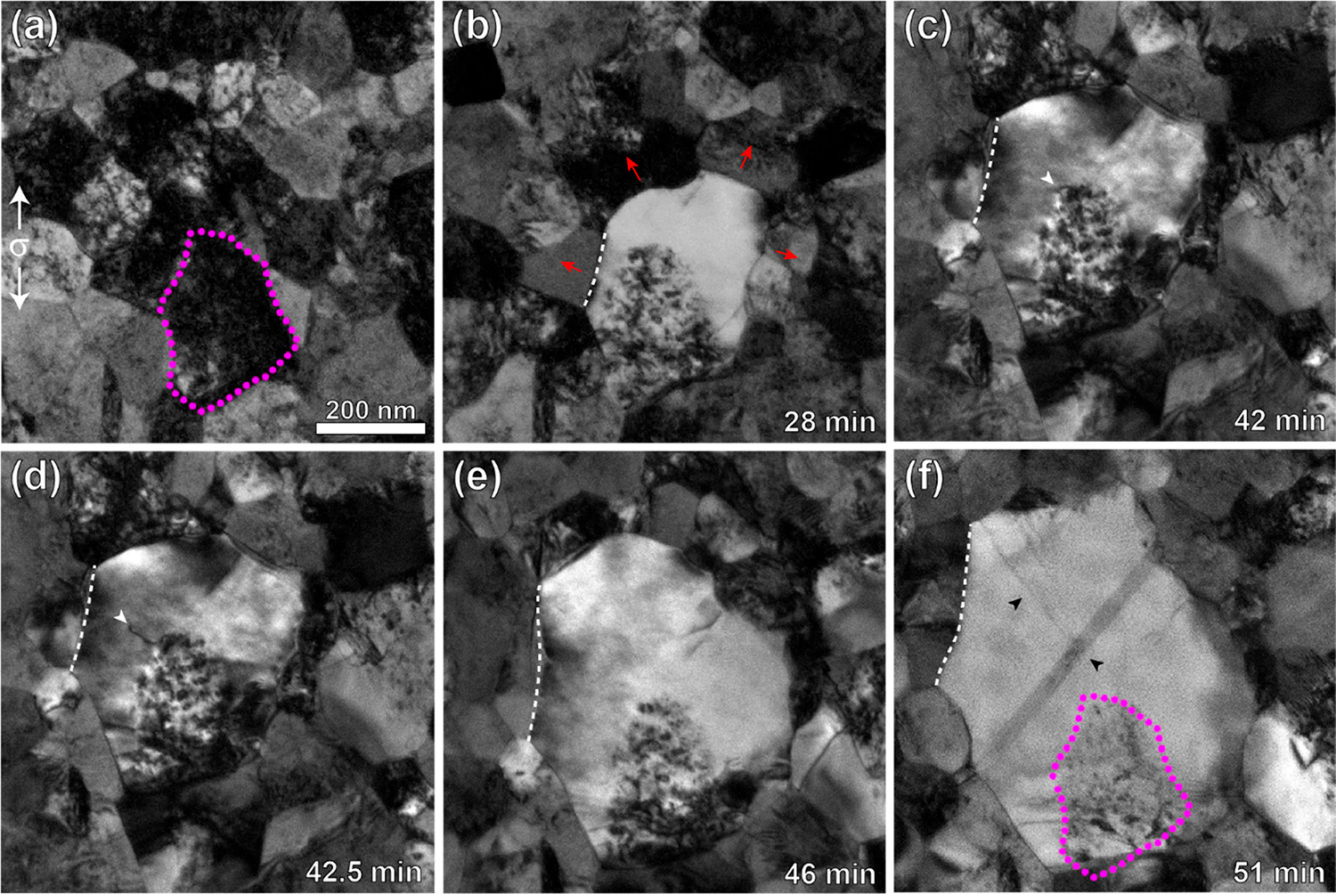
Stress-assisted GB migration leading to radiation damage healing and defect-free regions capable of supporting extensive dislocation glide. (a) Microstructure prior to an applied load with a single grain outlined. Direction of applied load (σ) indicated by vertical arrows. (b) GB migration has led to grain growth of the outlined grain resulting in defect-free regions where GB migration occurred. The radiation defects remain where the original grain was (no migrating GB passed through this region). Arrows indicate direction of continued GB migration. (c) GB migration continues and leads to a further increase in grain size and defect-free region. Arrow indicates a dislocation pinned on radiation defects. (d) The indicated dislocation becomes depinned and glides unrestricted in the radiation-free region until being absorbed by a nearby GB. (e) Continued GB migration leading to an increasing defect-free area. (f) The defect-free region can now support dislocation-dislocation interactions, indicated by both arrowheads. Time stamp in each indicates the total time (in minutes) under a tensile stress (both during loading and stress-relaxation).

The above results provide experimental evidence that a boundary migrating under an applied stress can effectively remove radiation damage, confirming previous models that stress-assisted GBM can lead to SFT absorption.^35−38^ It has been well documented that GBs absorb radiation defects under static conditions (owing to the increased radiation tolerance in NC/UFG metals), but the above results unambiguously highlight that a mechanical stress can activate an additional mechanism for damage removal. While GBs are known to act as diffusional sinks to radiation defects,^54^ this effect is typically localized to within nanometers of the GBs, much smaller than the tens of nanometers cleared of defects seen in the present study. The percentage of the area that is cleared of defects depends on the time under tension and stress levels (cleaned area percentage typically ranging between 2 and 15%). For example, 2.6% of the gauge area is cleared of radiation damage during the monotonic test shown in Figure 1e (total time under tension was 2.5 min) compared to the 14% that was cleaned after 51 min under tension in Figure 2. This indicates that pausing the loading prior to failure is necessary to allow for more time to promote and to observe the stress-assisted GBM and defect clearing. In this study, defect-free regions are always observed in the wake of a migrating GB, indicating that a wide range of GB types can absorb radiation defects during migration. This mechanism implies that the deleterious effects of irradiation on the mechanical properties of NC and UFG metals can be further reduced by an evolving network of migrating GBs when subject to mechanical loading. It is therefore crucial to quantify the effects of this healing mechanism on the evolving microstructure and resulting mechanical properties. Our quantitative *in situ* TEM technique is ideally suited, as it allows for direct quantification of microstructure evolution as a result of stress-assisted GBM with and without irradiation damage.

The effect of irradiation on GBM was further studied by comparing the GBM behavior for specific sets of grains in both irradiated and nonirradiated films. Figure 3 (Movie S7) is an example of such *in situ* TEM experiment consisting of successive stress-relaxation segments for an irradiated film where the outlined grain was tracked until specimen failure. The GBs marked 1 and 2 gradually migrate (red arrows indicate migration direction) while absorbing the radiation defects within the grain. This is clearly seen in Figure 3e, where the original grain outline is overlaid on the final microstructure to show the change in grain shape due to GBM. The majority of GB1 migrates at a steady velocity of 0.04–0.06 nm/s to a total migration distance of 83 nm (the right-most portion of this GB migrates an additional 31 nm at a maximum rate of 0.25 nm/s to result in the curved boundary seen in Figure 3e). The progression of GBs 1 and 2 migration, along with 4 other boundaries recorded simultaneously, can be seen in Figure 3f (curves for GB 1 and 2 are the gray and green curves, respectively). The average migration velocity (nm/s) for each tracked boundary is marked to the right of each curve. The instantaneous stress levels were measured and are displayed on the secondary axis. The stress ranged from 309 to 570 MPa with 22 instances of stress increase due to reloading in between relaxation segments (full stress–strain curve shown in Figure S7). For the majority of the boundaries, migration occurs at a relatively steady pace with average velocities ranging from 0.007 to 0.06 nm/s, which is consistent with the measured velocities for in Figure S5 at an applied stress of around 550 MPa. There is only one instance in which a GB jumped 16 nm at a maximum “jump” velocity of 8.1 nm/s. This is visualized by the large jump in migration distance in the brown data around 500 s and was associated with the collapsing of a smaller grain.

**Figure 3:**
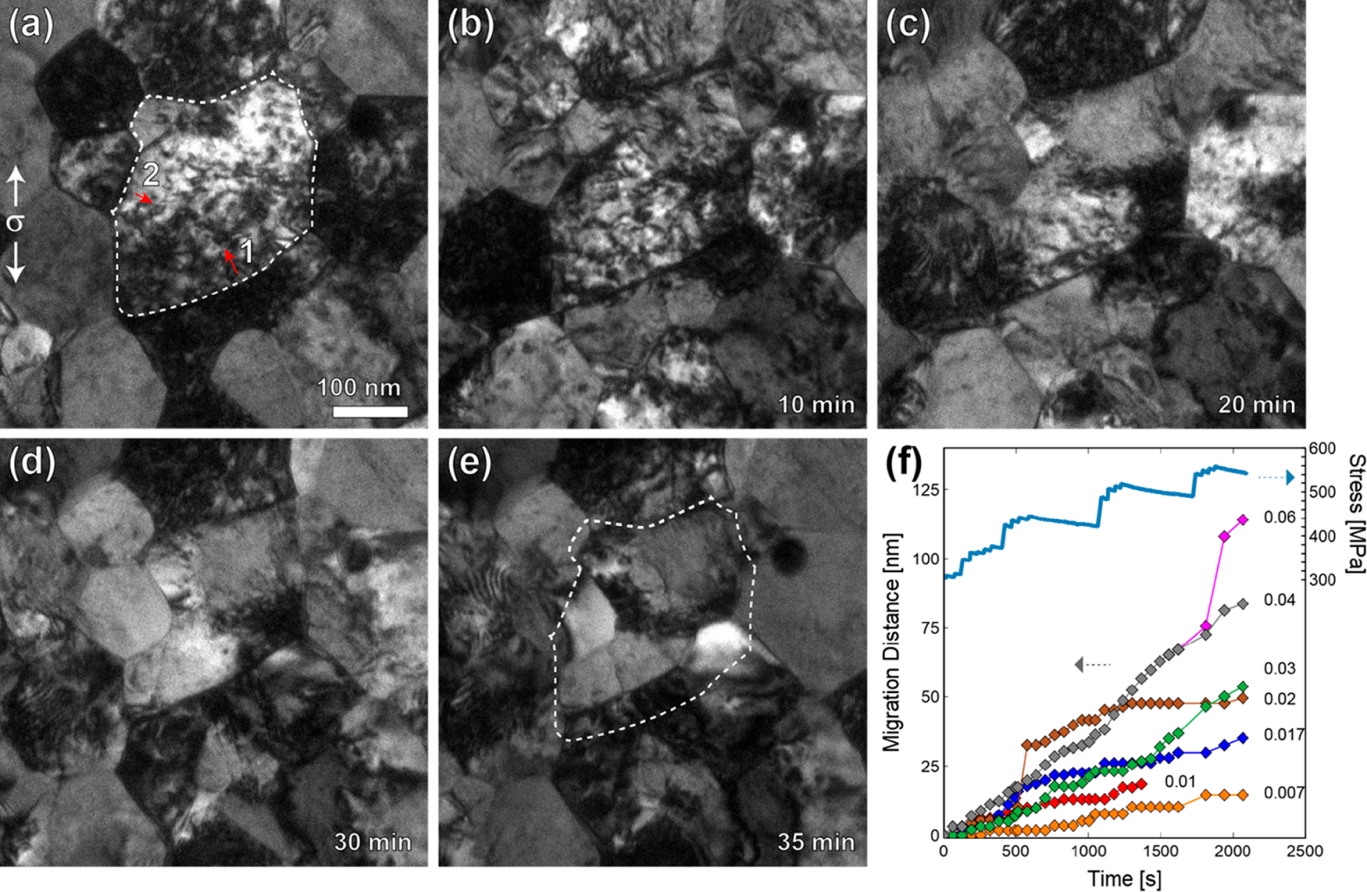
“Steady” grain boundary migration documented in irradiated film during repeated stress-relaxation experiment. (a) Microstructure prior to an applied load (in direction indicated by white arrows). The outlined grain is one of the grains tracked throughout the experiment with two migrating boundaries labeled 1 and 2. The red arrows indicate direction of GB migration. (b–d) the same grain shown in 10 min increments, (e) final microstructure with the original grain outline from part a overlaid to show the change in grain size and shape due to GB migration. The scale bar in part a is the same for all frames. (f) GB migration distance data throughout the experiment from six boundaries recorded simultaneously (data for GB 1 (gray/magenta) and GB 2 (green)). The instantaneous far-field stress levels are shown with the stress scale on the right *y* axis. Average velocity (nm/s) is recorded to the right of each curve. Time is given in terms of time since initial recording of the grains, which began 3 min after the initial load was applied.

A similar experiment was conducted on a nonirradiated specimen to identify the main differences in GBM for irradiated versus nonirradiated films. In general, the GBM occurs at a faster rate in the nonirradiated films which facilitates the GBs reaching a stable position within the time frame of an experiment. This is shown in Figure 4, where the outlined grain undergoes significant stress-induced GBM in the direction of the red arrows to result in the larger grain seen in Figure 4b,c with the final microstructure shown in Figure 4d (Movie S8). Migration data on these particular GBs as well as four additional migrating GBs are shown in Figure 4e. The total migration distances for the GBs associated with the growing grain in Figure 4a–d varied from 153 nm to a maximum migration of 270 nm over 25 min, with a maximum jump velocity of 85 nm/s corresponding to the first large jump in migration distance in the red line around 170 s. The four additional migrating GBs that were tracked simultaneously migrate quickly within the first few minutes and then stagnate, with migration velocities ranging from 0.03 to 0.12 nm/s (before stagnation). The instantaneous stress levels were not documented in this experiment, but the stress levels at different times were determined by measuring the displacement of the load sensor beams manually using TEM imaging and are indicated by the data points on the secondary stress axis in Figure 4e. The initial migration occurs at a far-field stress level of 340 MPa, with the stress recorded to vary between 197 to 367 MPa. The vertical gray dashed lines represent instances in which the stress is increased during a reloading segment. Half of the recorded GBs are sensitive to the loading and show an increase in migration speed directly after a reloading segment (vertical dashed line) which is followed by stagnation until the stress is increased again. The velocity of one of the GBs (red data) after the final three reloading segments until stagnation is 0.60, 0.22, 0.43 nm/s.

**Figure 4:**
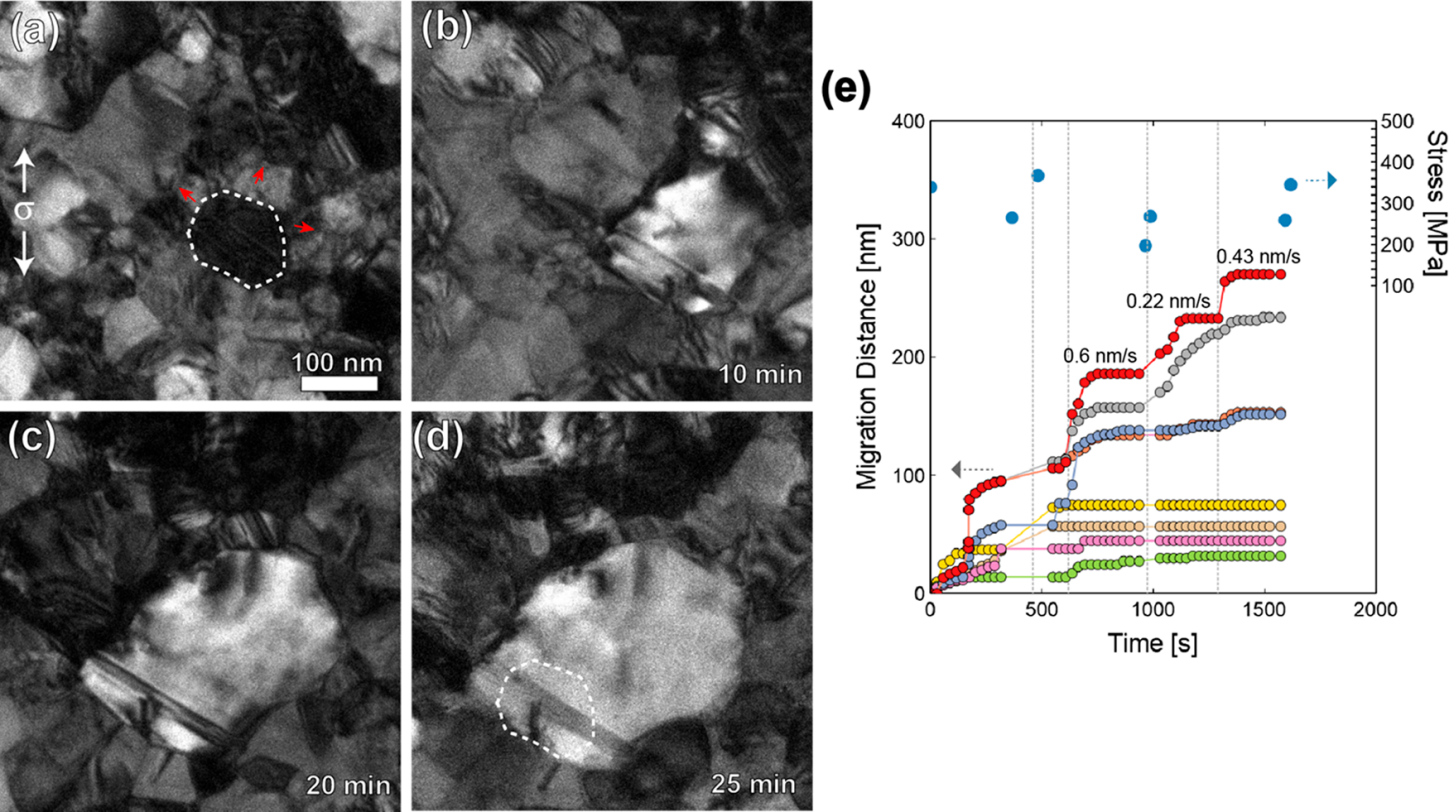
“Rapid” stress-induced grain boundary migration in nonirradiated films during a stress-relaxation experiment. (a) Initial microstructure of a collection of grains. The outlined grain undergoes significant grain growth due to GB migration in the direction indicated by red arrows. (b, c) The same grain in 10 min increments. (d) Final grain microstructure with original grain outline from part a overlaid to show the significant stress-induced grain growth. (e) GB migration distance for different boundaries tracked simultaneously. The GBs associated with the grain in parts a–d are shown in red, gray, orange, and light purple data. The manually measured stress levels are plotted on the secondary axis. Vertical gray lines represent reload instances in which the stress was increased. Time is given in terms of seconds since first recording of given set of grains which occurred 2.5 min after load was first applied. The velocity of the GB represented by the red data points is shown for the final three reloading segments (i.e., velocity of the GB as it migrates after promoted by an increase in stress until stagnation again).

Comparing the migration data (Figure 3f and Figure 4e) indicates that the majority of boundaries in the nonirradiated film migrate at faster velocities than the boundaries in the irradiated film. Analyzing the first 5 min only (Figure 5a) clearly shows that all of the tracked boundaries in the nonirradiated film experience faster migration leading to larger migration distances compared to the boundaries in the irradiated films. For example, the maximum migration velocity within the first 5 min in the irradiated film was 0.03 nm/s (gray data) whereas the migration velocities in the nonirradiated film varied from 0.04 to 0.30 nm/s despite the initial stress levels being comparable for both specimens. Figure 5b displays the “instantaneous” velocity for three GBs throughout the experiments shown in Figure 3 and Figure 4. Representative GBs were chosen to show the characteristic migration behavior for each specimen type. Comparing the velocity throughout the experiment clearly illustrates that the nonirradiated GBs (dashed) experience higher velocities before reaching stable equilibrium positions (i.e., velocity is zero). In contrast, the irradiated boundary (solid) continues migration at a relatively constant velocity. The instances of large velocity increase in the nonirradiated film occurring around 600, 970, and 1300 s corresponds to moments when the applied stress was reloaded to a higher value. The schematics shown in Figure 5, parts c and d, provide a visual comparison of the difference in GBM behavior in the grains analyzed in Figure 4 and Figure 3, respectively. The schematics show the original GB trace (solid) and subsequent GB trace outlines (dashed) in 10 min increments at the same length scale. The far-field applied stress ranges for the 20 min intervals are displayed below the schematics and indicate that the nonirradiated grains undergo stress-assisted GBM at larger velocities despite the fact that the applied stresses are on average lower than that in the irradiated films.

**Figure 5:**
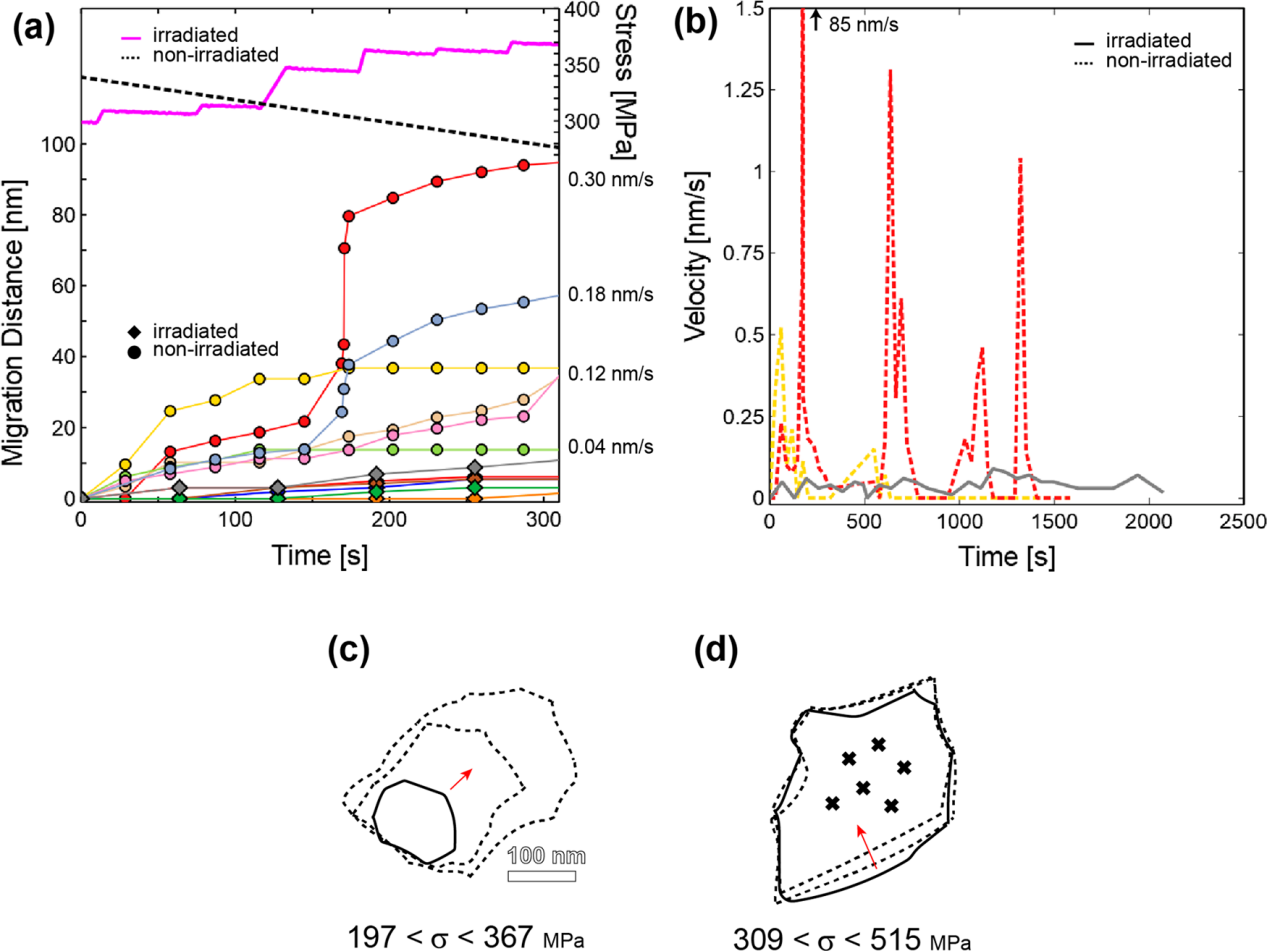
Comparison of stress-assisted grain boundary migration distances and velocity in irradiated and nonirradiated Au films. (a) GB migration distance data from the irradiated film in Figure 3 (diamonds) and nonirradiated specimen in Figure 4 (circles) scaled to show first 5 min only. The stress levels for both are shown with the stress scale on the right *y* axis to show that the initial stress levels are similar followed by an increase in stress in the irradiated film and decrease in stress in nonirradiated. The average velocity is provided for each nonirradiated GB showing a range of 0.04–0.30 nm/s. (b) “Instantaneous” velocity for one GB in irradiated specimen (Figure 3) and two GBs in nonirradiated specimen (Figure 4). Each GB was chosen to display characteristic behavior for each specimen type. “Instantaneous” velocity is defined as the velocity for a 30–60 s interval. The schematic of the grain shape change outline of (c) nonirradiated grain in Figure 4 and (d) irradiated grain in Figure 3 to compare migration behavior at the same length scale (scale bar the same for both). The solid outline represents the initial grain size with the two dashed outlines representing the grain shape after 10 and 20 min. The red arrow indicates direction of migration. The black “×” represents radiation damage. The stress ranges during migration are provided underneath the schematics.

Using the GB velocity information shown in Figures 3– 5 to conclude the effect of irradiation on GB mobility would require knowledge of the driving force for GBM (since velocity is the product of mobility and driving force), which is challenging to quantify accurately.^55−57^ Qualitatively, we believe that the increased GB velocities for the as-deposited, unirradiated Au films are related to an increased driving force associated with the smaller grains that are only present in these films. As stress is applied, small grains deform elastically whereas larger grains achieve lower stresses by deforming plastically. The size-dependent yield stress has been proposed as a size-dependent driving force for grain coarsening and can explain the specific observation that large grains (with lower strain energy densities) grow while small grains (with higher strain energy densities) shrink and disappear.^58,59^ This explanation is consistent with our observations of “rapid” grain growth for the unirradiated Au films associated with the disappearance of the smaller grains. In addition, “rapid” grain growth is not observed for unirradiated, *annealed* specimens (350 °C for 30 min) that do not have small grains (<50 nm) initially present (Figure S8a,c), which is also consistent with the notion that small grains are associated with larger driving force for GBM (and therefore increased velocities). Given the absence of small grains in irradiated samples and nonirradiated annealed samples, comparing the average GBM velocities (Figure S8b) suggests that the irradiated GBs have lower mobilities (similar velocities, larger applied stresses for the irradiated specimen). Lower GB mobilities could be attributed to increased disorder of the GB plane as the GB absorbs the radiation defects that could make disconnection motion more “sluggish”, or that the GBs are being temporarily pinned by the radiation defects. Additional experiments, particularly on irradiated annealed specimens, are required to further investigate the effect of irradiation on GB mobility.

It is important to note that the observed GBM behavior in the irradiated films exhibit similarities with dynamic recrystallization, which is typically observed in terms of newly nucleated (defect-free) grains growing at the expense of neighboring grains with high defect density.^60^ However, we observe stress-assisted GBM with the unirradiated films, indicating that migration occurs without the additional defect-removal driving force and therefore it is likely that stress can also trigger GBM in irradiated films, as suggested in.^35^ Furthermore, in Figure 2, the grain that grew was not a small defect-free embryo, but instead had a high density of defects similar to the surrounding grains. In conclusion, this study provides striking evidence that stress-assisted GBM serves as an effective mechanism of defect removal in irradiated NC and UFG metals. This indicates that applying a small stress (below the yield stress) sufficient enough to promote GBM could be implemented to facilitate “self-healing” of irradiated materials at the expense of moderate plastic deformation. Future studies will involve characterizing the stress-assisted GBM in detail, including at elevated temperatures, with particular interest in understanding how the irradiation-induced defects influence the GB structure and the GBM behavior in order to optimize the defect clearing capacity of NC and UFG metals.

## Supporting Information Available

Specimen preparation and irradiation, defect spacing measurement, in situ TEM nanomechanical testing, grain boundary migration in annealed UFG Au (PDF)
Movie S1. Dislocation pinning on radiation damage during straining (corresponding to Figure S4) (video speed: 5×) (AVI)
Movie S2. Intragranular plasticity (dislocation pinning) during straining (video speed: 5×) (AVI)
Movie S3. Stress-induced grain boundary migration. One portion of the microstructural evolution of the grain shown in Figure 2 (video speed: 5×) (AVI)
Movie S4. Continued microstructure evolution during deformation corresponding to the grain in Figure 2, including dislocation pinning and de-pinning, followed by grain boundary migration leading to an increasing area free of defects (video speed: 5×) (AVI)
Movie S5. Stress-assisted grain boundary migration leads to defect free region that can now support extended dislocation glide and dislocation–dislocation interactions (Figure 2f) (video speed: 5×) (AVI)
Movie S6. Stress-assisted grain boundary migration and defect absorption with evidence of disconnection flow within boundary (video speed: 5×) (AVI)
Movie S7. Steady stress-assisted grain boundary migration in irradiated film corresponding to the grain in Figure 3 (video speed: 10×) (AVI)
Movie S8. Rapid stress-assisted grain boundary migration in non-irradiated film (Figure 4) (video speed: 10×) (AVI)

## Supplementary Material

nl3c00180_si_001.pdf

nl3c00180_si_002.avi

nl3c00180_si_003.avi

nl3c00180_si_004.avi

nl3c00180_si_005.avi

nl3c00180_si_006.avi

nl3c00180_si_007.avi

nl3c00180_si_008.avi

nl3c00180_si_009.avi
