# Supplementary material for: Direct Observation of Grain-Boundary-Migration-Assisted Radiation Damage Healing in Ultrafine Grained Gold under Mechanical Stress
Source: Nano Lett. 2023 Apr 14;23(8):3282–90. doi: 10.1021/acs.nanolett.3c00180 (PMC10141400; doi:10.1021/acs.nanolett.3c00180)
Supplement: Supplementary file 1 — nl3c00180_si_001.pdf [file nl3c00180_si_001.pdf]

Supporting Information for

Direct observation of grain-boundary-migration-assisted radiation damage healing in ultrafine grained Au under mechanical stress

Sandra Stangebye[a], Kunqing Ding[b], Yin Zhang[b], Eric Lang[c], Khalid Hattar[d,e], Ting Zhu[b], Josh Kacher[a], Olivier Pierron[b*]

[a] School of Materials Science and Engineering, Georgia Institute of Technology, Atlanta, GA 30332, USA

[b] Woodruff School of Mechanical Engineering, Georgia Institute of Technology, Atlanta, GA 30332, USA

[c] Nuclear Engineering Department, UNM, Albuquerque, NM 87131, USA

[d] Sandia National Laboratories, Albuquerque, NM 87185, USA

[e] Department of Nuclear Engineering, University of Tennessee, Knoxville, TN 37996, USA

Specimen preparation and Irradiation. The 100 nm-thick nanocrystalline (NC) and ultra-fine grained (UFG) Au specimens were electron beam evaporated onto a patterned wafer followed by lift-off and final etching of the Si substrate to reveal dog-bone shaped tensile specimens (specimen fabrication details can be found in Ref. [1]). A 2.8 MeV Au^{4+} ion beam was used with a fluence of 5.5×10^{13} ions cm^{-2}. Based on the Stopping and Range of Ions in Matter (SRIM 2013) [2] (Kinchin-Pease Estimates) using a 40 eV displacement energy [3], **Figure S1** shows the average dpa in the Au film is ~.65 dpa (displacement per atom), and there is minimal Au implantation in the film, with the peak implantation concentration of 30 appm. Grain sizes are determined as the equivalent diameter for a circle of the same area as that particular grain and grain size averages are reported as area average.

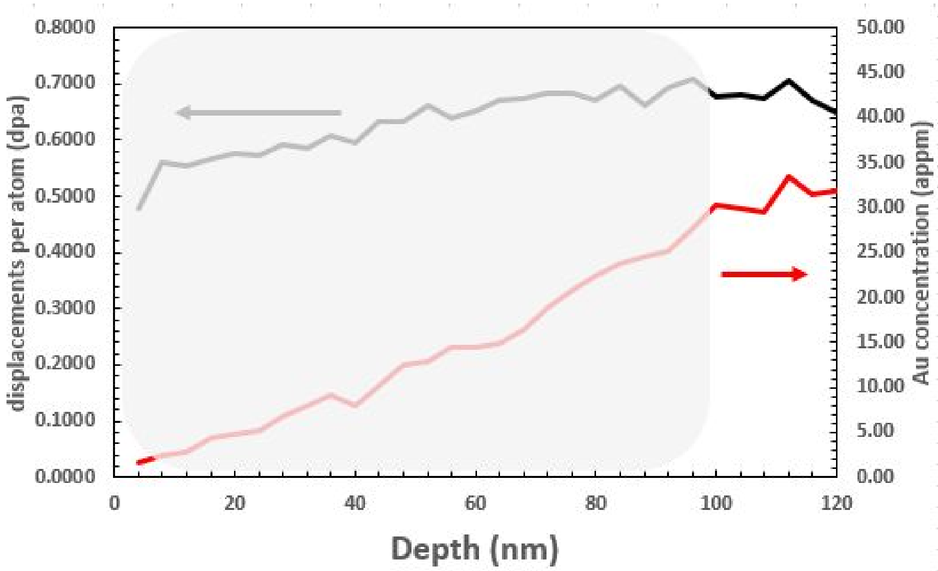

Figure S1. SRIM-estimated damage profile (black) and Au implantation profile (red) for 100 nm-thick Au film irradiated with 2.8 MeV Au ions. Shaded area in the plot indicates the thickness of the Au film, offering a visual guide for readers. Average dpa in the film is ~0.65 dpa and peak implantation level is 30 appm.

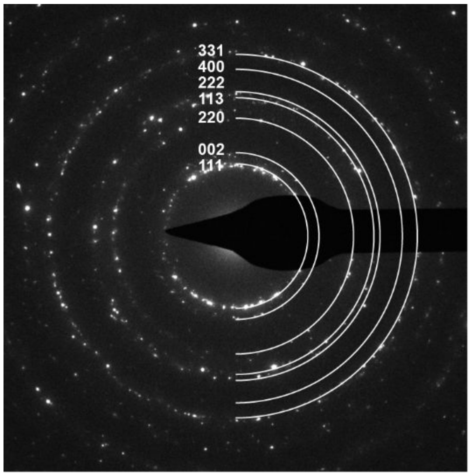

Figure S2. Indexed diffraction pattern of irradiated Au films exhibiting characteristic ring structure for NC and UFG FCC metals.

Defect Spacing Measurement. The defect spacing between radiation defects was estimated using the following approach. Weak-beam dark field (WBDF) TEM images were taken of the initial irradiated microstructure. From the WBDF image, defects were individually identified and counted. For the grain shown in **Figure 1**c, the parameters in calculating the defect spacing are shown in **Table S1**. An invisibility criterion of 1.9 was used to estimate the defects that are invisible at any the particular diffraction condition [3].

Table S1. Parameters used in estimating defect spacing.

Number of counted defects	614
Total number of defects	1166
Grain area (nm^2)	42732
Defect density ρ (nm^{-3})	2.73×10^{-4}

The final defect spacing l was estimated as ~15 nm from the defect density ρ using the following equation:

$$l = \frac{1}{\sqrt[3]{\rho}}$$

The above equation assumes evenly spaced defects and does not consider the small portion (~5nm) of the thickness that is denuded of defects near either free-surface. However, if we assume the radiation defects are SFT, then there are minimal diffraction conditions in which the defects are completely invisible. If this assumption is correct, no invisibility criterion is used and the defect spacing only increases to ~19 nm.

***In situ* TEM nanomechanical testing.** The *in situ* tests were conducted using a custom micro-electromechanical system (MEMS) device that utilizes capacitive sensing for stress and strain quantification. The operation of the MEMS device, shown in **Figure S3**a, involves applying a voltage across the thermal actuator (TA) which imparts a displacement of the central shuttle

through resistive heating. This displacement causes a shift in the mobile beams in the capacitive sensors (CS_1 and CS_2) which leads to a measured change in capacitance for each sensor. Treating the capacitive sensors as parallel plate capacitors, the displacement of each sensor (X) can be calculated based on the change in capacitance following:

$$C = \alpha\kappa\varepsilon_0 nA\left[\left(\frac{1}{d_1 - X} + \frac{1}{d_2 + X}\right) - \left(\frac{1}{d_1} + \frac{1}{d_2}\right)\right]$$

where α is the calibration constant, κ is the relative permittivity of air, $\varepsilon_0 = 8.854 \times 10^{-12}\ \mathrm{Fm}^{-1}$ is the permittivity of free space, n is the number of comb structures (n = 42), A is the overlapping area of the comb structure, and d_1 and d_2 are the nominal gaps in the comb structure. CS_1 is rigidly connected to the TA and thus measures the displacement of the actuator (X_A) and CS_2 is connected to the load sensor beams and measures the displacement of the load sensor (X_{LS}). From these displacements, the displacement of the specimen X_S and the force on the specimen (F) can be determined:

$$X_S = X_A - X_{LS}$$

$$F = K_{LS} X_{LS}$$

Then, using the initial specimen geometry, the stress and strain can be calculated. A SEM image of a tensile specimen on the MEMS device is shown in **Figure S3**b. The specimens are manipulated onto the MEMS using a micromanipulator under a light microscope and clamped using UV curable epoxy. In cases when the capacitive sensors were not operating correctly, the stress level can be determined by manually measuring the deflection of the load sensor beams. The capacitive sensing has a typical noise level of 0.1-0.2 fF in capacitance which translates to a precision of 1-2 MPa in stress. The image-based sensing has improved noise levels of ~ 0.1 nm (< 0.1 MPa).

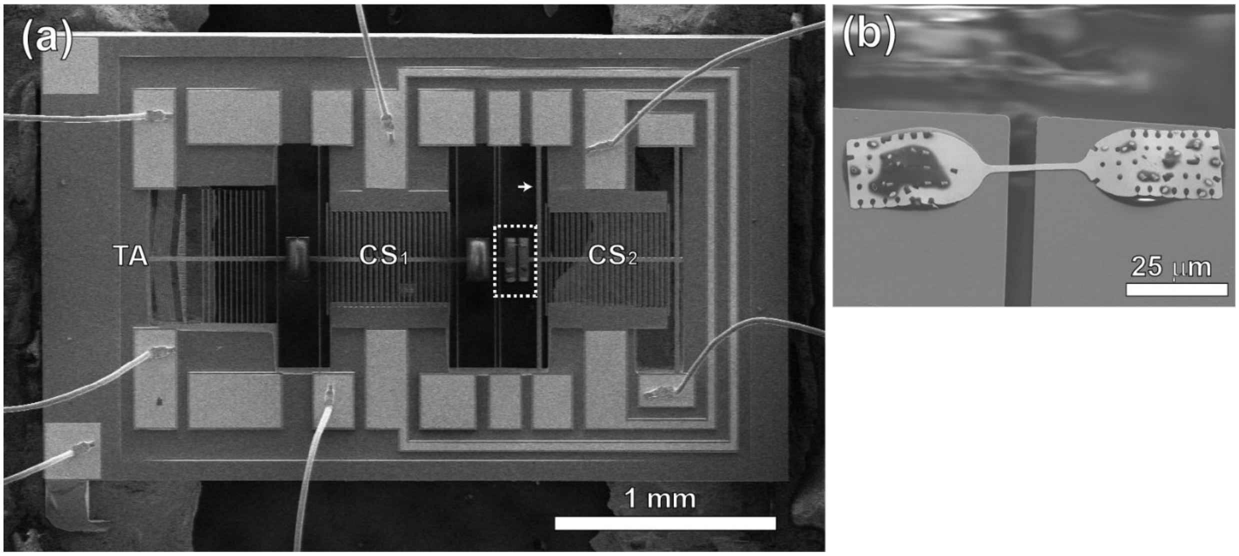

Figure S3. SEM image of (a) MEMS device used to conduct *in situ* TEM nanomechanical experiments. The main components are labeled: thermal actuator (TA), capacitive sensor one (CS1), capacitive sensor two (CS2), specimen gap (dashed box) and load sensor beams (arrowed), (b) tensile specimen clamped to specimen gap with UV curable epoxy.

The experiments are conducted in an FEI Tecnai F30 TEM operating at an accelerating voltage of 300 kV. TEM micrographs and videos were recorded using a Gatan OneView camera. Snapshots taken from the recorded videos are overlaid and GB migration distances were measured manually using Adobe Photoshop. In some experiments, the MEMS displacement is gradually increased (as in regular monotonic tests to failure) but stopped at various points to allow for longer observations before failure occurs. During these holds, the applied stress relaxes (since plastic deformation occurs over time), leading to stress relaxation.

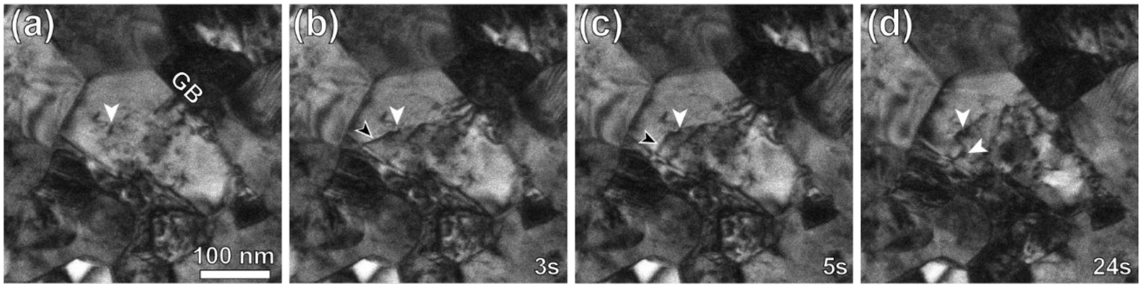

Figure S4. Radiation defects serving as obstacles to dislocation glide. (a) Grain containing multiple radiation-induced defects with one specific defect indicated by white arrowhead. The GB active in this example is labeled accordingly. (b) After 3 seconds, dislocations are emitted from the indicated GB and partially transverse the grain until being pinned by defects. One pinned dislocation (indicated by black arrowhead) is pinned by the defect that is indicated by white arrowhead in both (a) and (b) and the leftmost GB. (c) After two additional seconds, the left end of the dislocation (black arrowhead) glides while a portion remains pinned by a defect (white arrowhead). (d) a portion of the dislocation continues to glide until being pinned by an additional defect indicated by the second white arrowhead. The accompanying video is Supplementary Movie 2.

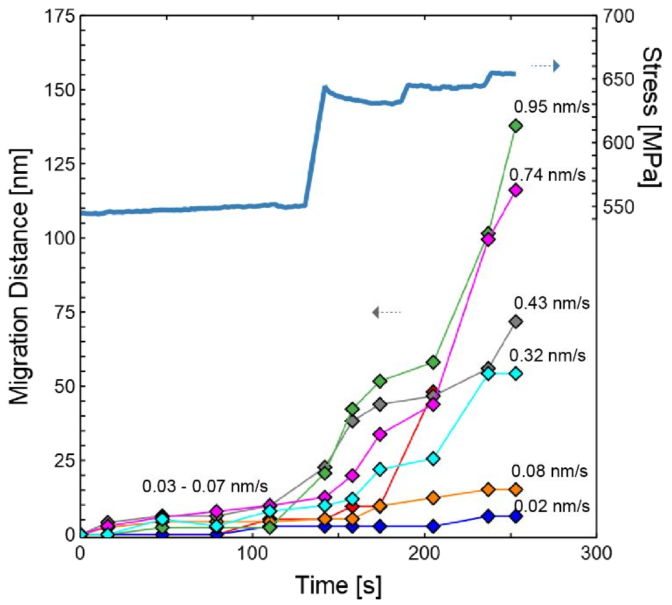

Figure S5. GB migration distance data for all the involved boundaries during the transition from **Figure 2**c-f and Supplementary Movie 4. The far-field stress values are provided on the secondary axis to demonstrate how migration behavior varies with stress. The first 130 seconds of the data correspond to a stress-relaxation segment and during this time the average velocities for all the GBs ranged between 0.03-0.07 nm/s (displayed on graph). After this, the stress is reloaded to a higher level (650 MPa) and the migration distance/velocity for most of the boundaries increase accordingly. The average velocity of each boundary after the stress reload at 130 seconds is provided to the right of the curves.

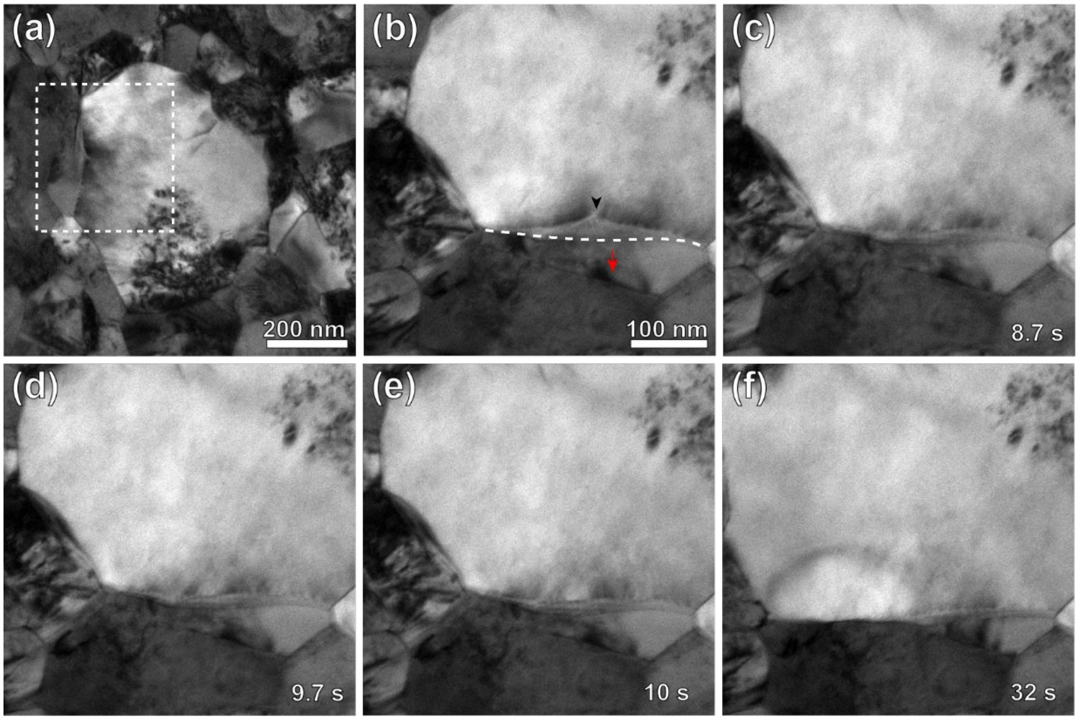

Figure S6. Grain boundary migration facilitated by disconnection flow within boundary. (a) Low magnification to show the full grain being documented. The dashed box indicates the region that (b)-(f) are focused on. (b) A magnified view of one of the active GBs that migrates outward in the direction of the red arrow (note perspective has been rotated 90° counterclockwise). The grain boundary is not edge-on which allows for view of disconnection flow (indicated by variations in contrast) in the boundary. A kink in the boundary is indicated by the black arrowhead. (c)-(f) progression of the GB migration at an average velocity of 0.68 nm/s. Time is

provided in terms of time since (b). Movie of progression is Supplementary Movie 6.

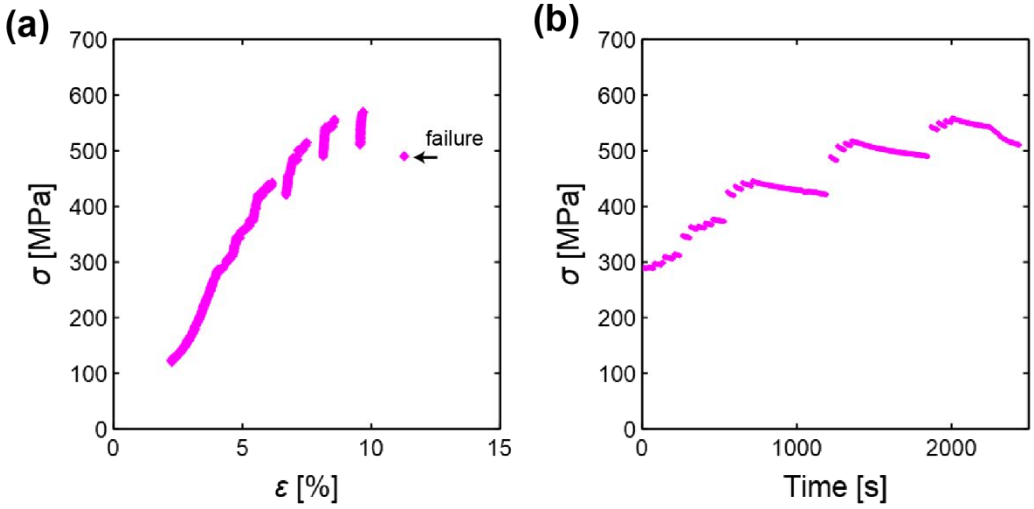

Figure S7. Stress-relaxation data for specimen shown in **Figure 3**. (a) Full stress-strain curve until specimen failure. Kinks or discontinuities correspond to when the loading was paused and the stress relaxes (i.e. stress-relaxation segments). (b)The stress-relaxation segments. This is the same data shown in **Figure 3**f (right *y* axis) but includes the relaxation segment only (no loading segments)

Grain boundary migration in annealed UFG Au. A portion of the UFG unirradiated specimens were annealed to investigate how the observed stress-induced GB migration behavior varies when the smallest grains are removed prior to mechanically loading. The specimens were annealed at 350 °C for 30 min which resulted in an average grain size of 287 nm, with the majority of small grains ($d < 50$ nm) removed. The initial microstructure is shown in **Figure S8**a. A stress-relaxation experiment, with four relaxation segments, was conducted and GB migration was tracked with stress $\sigma \sim 260 - 350$ MPa. The migration data for the annealed specimen is shown below in **Figure S8**b and c compared with the migrating boundaries in the irradiated (**Figure 3**) and non-irradiated (**Figure 4**), respectively. GB migration behavior of the unirradiated annealed specimens is similar to that in the irradiated, with average velocities ranging from 0.01-0.03 nm/s and no occurrence of rapid increase in GB migration. These velocities are similar to the average velocities of the migrating GBs in the irradiated films within the first 5 minutes (**Figure 5**a) for a similar stress range of $309 - 370$ MPa. Considering that the stress range in the annealed film ($260 - 350$ MPa) is similar to the stress range for the as-deposited specimen in **Figure 4** ($197 - 367$ MPa) indicates that the slower migration velocities in the annealed specimen is likely due to the prior removal of the unstable, small grains from the annealing processes.

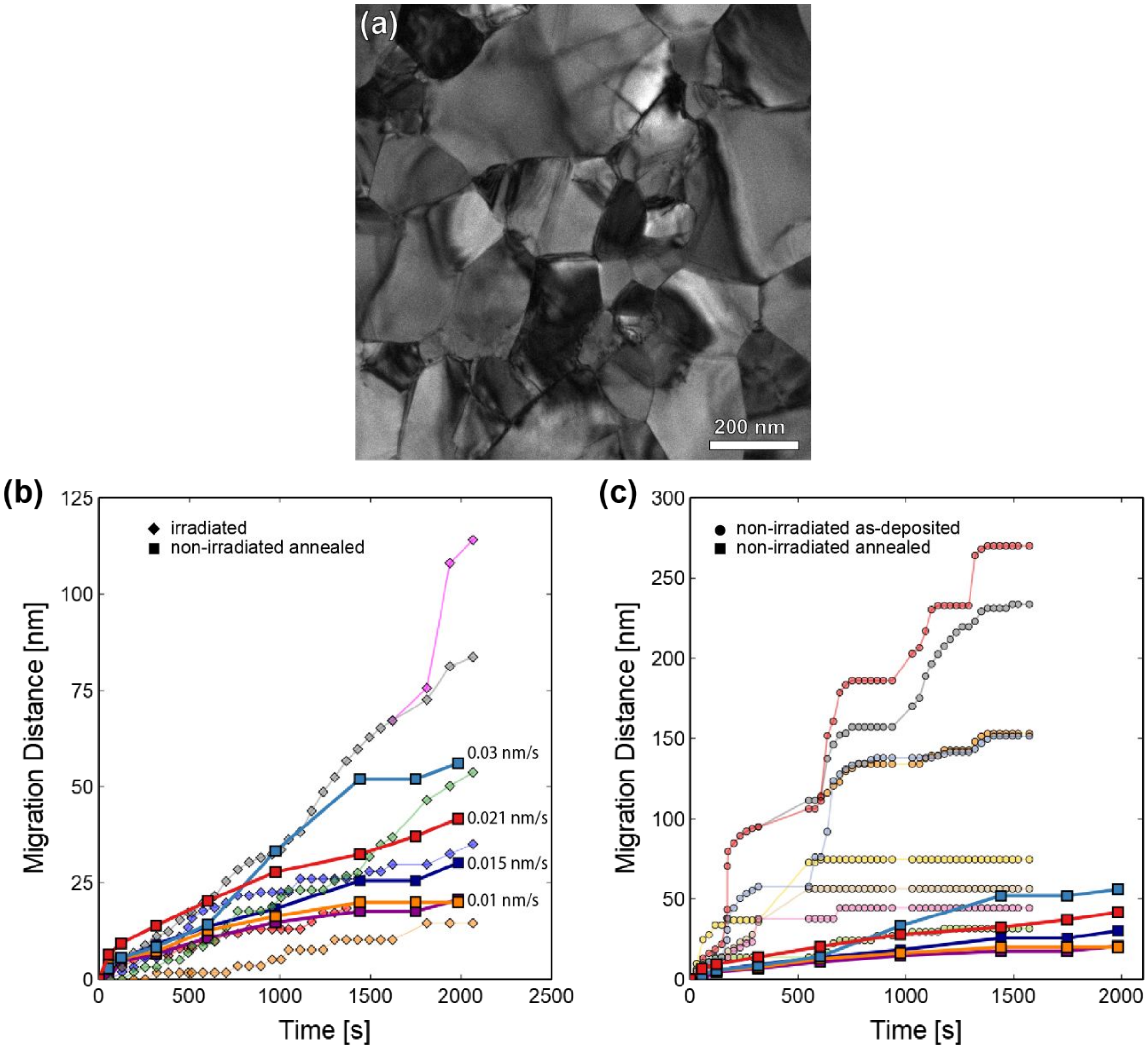

Figure S8. GB migration data for migrating boundaries in a UFG annealed Au specimen. (a) TEM image of initial microstructure of UFG annealed Au. Migration behavior of annealed Au compared with the migrating boundaries in (b) irradiated specimen (**Figure 3**) and (c) non-irradiated as-deposited (**Figure 4**). Average velocity for the annealed GBs are 0.01, 0.015, 0.021, and 0.03 nm/s (displayed to the right of each curve in (b)). Results indicate that migration behavior (distance and average velocity) is similar for irradiated and unirradiated annealed Au. Stress ranges for each data set: annealed $(260 - 350\ \mathrm{MPa})$, irradiated $(309 - 570\ \mathrm{MPa})$, non-irradiated $(197 - 367\ \mathrm{MPa})$.

Supplementary Movie Captions

Movie 1. Dislocation pinning on radiation damage during straining (corresponding to **Figure S4**). Video speed: 5x.

Movie 2. Intragranular plasticity (dislocation pinning) during straining. Video speed: 5x.

Movie 3. Stress-induced grain boundary migration. One portion of the microstructural evolution of the grain shown in **Figure 2**. Video speed: 5x.

Movie 4. Continued microstructure evolution during deformation corresponding to the grain in **Figure 2**, including dislocation pinning and de-pinning, followed by grain boundary migration leading to an increasing area free of defects. Video speed: 5x.

Movie 5. Stress-assisted grain boundary migration leads to defect free region that can now support extended dislocation glide and dislocation-dislocation interactions (**Figure 2**f). Video speed: 5x.

Movie 6. Stress-assisted grain boundary migration and defect absorption with evidence of disconnection flow within boundary. Video speed: 5x.

Movie 7. Steady stress-assisted grain boundary migration in irradiated film corresponding to the grain in **Figure 3**. Video speed: 10x.

Movie 8. Rapid stress-assisted grain boundary migration in non-irradiated film (**Figure 4**). Video speed: 10x.

SI References

[1] E. Hosseinian, O.N. Pierron, Quantitative in situ TEM tensile fatigue testing on nanocrystalline metallic ultrathin films, Nanoscale. 5 (2013) 12532–12541.

[2] R.E. Stoller, M.B. Toloczko, G.S. Was, A.G. Certain, S. Dwaraknath, F.A. Garner, On the use of SRIM for computing radiation damage exposure, Nucl. Instruments Methods Phys. Res. Sect. B. 310 (2013) 75–80.

[3] K. Nordlund, A.E. Sand, F. Granberg, S.J. Zinkle, R. Stoller, R.S. Averback, T. Suzudo, L. Malerba, F. Banhart, W.J. Weber, F. Willaime, S. Dudarev, D. Simeone, Primary Radiation Damage in Materials, 2015.
